# Delayed Appendectomy Versus Early Intervention: A Systematic Review of Outcomes in Complicated Appendicitis

**DOI:** 10.7759/cureus.94868

**Published:** 2025-10-18

**Authors:** Nazim F Hamed, Turki Dakhil Alofi, Mohammad Yasir Alamori, Sarah Fahad Alkhan

**Affiliations:** 1 General Pediatrics, Security Forces Hospital, Dammam, SAU; 2 Pediatric Surgery, Security Forces Hospital, Dammam, SAU; 3 Medicine, Security Forces Hospital, Dammam, SAU

**Keywords:** complicated appendicitis, delayed surgery, early surgery, management, systematic review

## Abstract

The management of complicated appendicitis remains a subject of debate, particularly concerning the optimal timing of surgical intervention. While early appendectomy has been the traditional approach, there is growing clinical interest in delayed surgery with initial antibiotic therapy. The existing evidence, however, presents conflicting outcomes, creating uncertainty for surgeons. Therefore, the rationale for this study is to synthesize the available evidence to provide a clearer understanding of the comparative outcomes of early versus delayed surgical strategies. This systematic review aims to evaluate the outcomes of delayed versus early surgical intervention in patients with complicated appendicitis. A comprehensive search of four databases yielded 452 relevant publications. After duplicate removal using Rayyan QCRI, 162 records were screened for relevance, and 29 full-text articles were assessed for eligibility. Ultimately, three studies met the inclusion criteria for evidence synthesis. The total pooled population was 41,274 patients (Early Surgery [ES] Group: 35,923; Delayed Surgery [LS] Group: 5,351), of whom 22,789 (55.2%) were male. The analysis revealed a nuanced tradeoff between the two approaches. Early surgery was consistently associated with a shorter overall hospital stay and lower medical costs. However, it was linked to a higher incidence of wound infections. Conversely, delayed surgery was associated with a significantly lower rate of major surgical procedures, such as ileocecectomy or right hemicolectomy, and fewer overall complications, despite sometimes resulting in a longer hospitalization. These findings highlight the complexity of decision-making in complicated appendicitis. The choice between early and delayed intervention involves balancing the risks of infection against the need for more extensive surgery. This contradiction in the literature underscores the necessity for further rigorous, prospective studies to definitively establish optimal timing and develop tailored treatment strategies that improve patient outcomes.

## Introduction and background

Acute appendicitis is one of the most common surgical abdominal emergencies, with an estimated lifetime incidence of 6.7% in women and 8.6% in men. It predominantly affects a young population and typically presents as lower abdominal pain [1,2]. In a significant subset of patients, the condition progresses to complicated appendicitis, which is characterized by perforation, abscess, or phlegmon formation. Identifiable risk factors for this progression include age over 50, female gender, symptom duration exceeding 48 hours, a high Alvarado score, CRP levels greater than 100 mg/L [3-6], and an elevated risk of surgical-site infections in diabetic patients [7]. Annually, over 300,000 appendectomies are performed, with approximately 20% of cases involving these serious complications [8]. The diagnostic and therapeutic challenges are often compounded by delays in seeking medical care, making the management of complicated appendicitis a persistent subject of clinical debate [9,10].

While the necessity of surgical intervention is unequivocal, the optimal timing for an appendectomy in these complex cases remains a significant point of contention. Emerging evidence suggests that the timing of surgery is a critical determinant of patient outcomes, influencing postoperative morbidity, the duration of hospitalization, and overall recovery [11,12]. Although early appendectomy has been the conventional approach to prevent disease progression, a growing body of literature proposes that a delayed strategy, often involving initial antibiotic therapy and/or percutaneous drainage, may reduce the incidence of major surgical procedures and certain complications without compromising patient safety. This divergence between traditional practice and contemporary research underscores the need for a comprehensive synthesis of the available evidence.

Therefore, this systematic review aims to synthesize the current evidence to directly compare the outcomes of early versus delayed appendectomy in patients with complicated appendicitis. It will specifically evaluate critical metrics, including postoperative complication rates, the length of hospital stay (LOS), the need for more extensive surgery, and overall healthcare utilization.

## Review

Methods

This systematic review was conducted in strict accordance with the Preferred Reporting Items for Systematic Reviews and Meta-Analyses (PRISMA) guidelines to ensure transparency and reproducibility. The primary objective was to compare the outcomes of early versus delayed surgical intervention for complicated appendicitis.

Search Strategy and Data Sources

A comprehensive literature search was performed across four electronic databases: PubMed, Web of Science, Scopus, and ScienceDirect. The search was conducted for articles published from January 2002 to June 2025. The search strategy utilized a combination of Medical Subject Headings (MeSH) terms and keywords, including "complicated appendicitis," "perforated appendicitis," "appendiceal abscess," "early appendectomy," "delayed appendectomy," "interval appendectomy," "appendectomy timing," "postoperative complications," "length of stay," and "treatment outcomes." These terms were combined using Boolean operators (AND, OR) to maximize the retrieval of relevant studies.

Eligibility Criteria

Inclusion criteria: The inclusion criteria are listed as follows:

Study designs: Randomized controlled trials (RCTs), cohort studies, and case-control studies.

Participants: Patients of any age or gender with a diagnosis of complicated appendicitis (e.g., perforation, abscess, or phlegmon).

Intervention and comparison: Studies directly comparing "early" versus "delayed" appendectomy. For this review, "early appendectomy" was defined as surgery performed within 24 hours of diagnosis or hospital admission. "Delayed appendectomy" was defined as initial non-operative management (e.g., antibiotics with or without percutaneous drainage) followed by scheduled surgery, typically 6-8 weeks later.

Outcomes: Studies must report on at least one of the following: postoperative complication rates, LOS, need for major surgery (e.g., ileocecectomy), or healthcare costs.

Publication: Articles published in English within the last 20 years.

Exclusion criteria: Studies were excluded if they were review articles, editorials, case reports, or conference abstracts; focused solely on simple appendicitis; did not provide a clear comparison based on surgical timing; had insufficient or duplicated data; or failed to provide clear definitions for the timing of interventions.

Study Selection and Data Extraction

The study selection process was managed using the Rayyan QCRI web tool (Rayyan Systems Inc., Cambridge, MA). After removing duplicates, two independent reviewers screened titles and abstracts against the eligibility criteria. The full texts of potentially relevant studies were then retrieved and assessed in detail. A study was excluded at the full-text stage only if it definitively did not meet the pre-specified inclusion criteria, with reasons for exclusion documented. Any disagreements between reviewers were resolved through consensus or consultation with a third reviewer.

Data from the included studies were extracted using a standardized form, capturing details on study characteristics (author, year, design), patient demographics, definitions of early/delayed groups, and relevant outcomes.

Data Synthesis and Quality Assessment

A meta-analysis was not deemed appropriate due to the anticipated clinical and methodological heterogeneity among the included studies (e.g., variations in patient populations and precise definitions of outcomes). Therefore, a qualitative synthesis was planned, with findings summarized in narrative and tabular forms to compare the outcomes of early versus delayed intervention.

The risk of bias in the included non-randomized studies was assessed by two independent reviewers using the ROBINS-I (Risk Of Bias In Non-randomized Studies - of Interventions) tool [13]. This tool was selected for its rigor in evaluating confounding and other biases prevalent in observational studies. Any discrepancies in quality assessment were resolved through discussion.

Results

**Figure 1:**
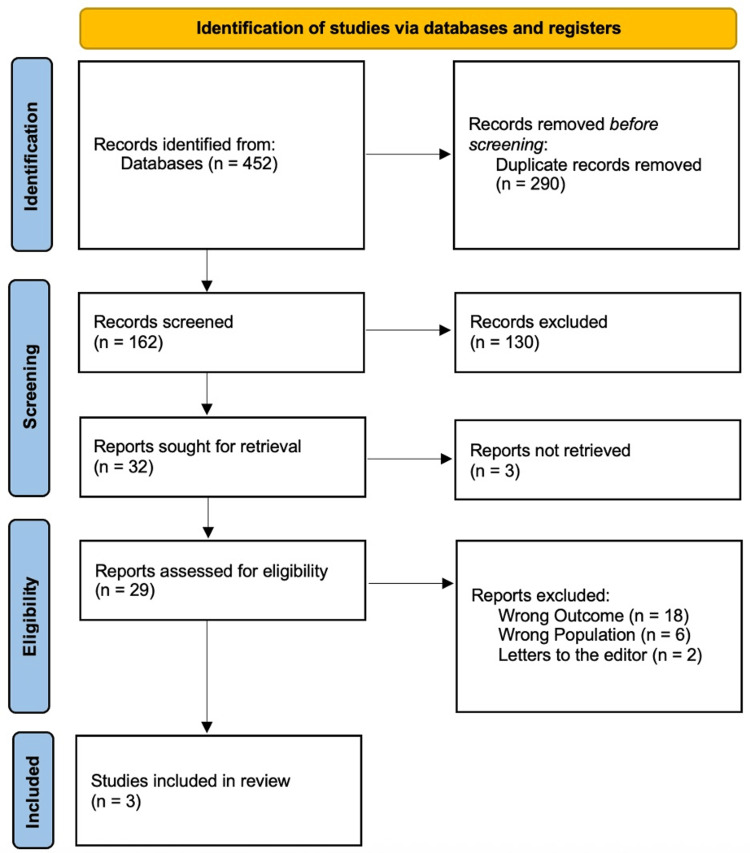
PRISMA flowchart Ref. [14].

Figure 1 shows that 452 items were found using the designated search method. One hundred and sixty-two articles were assessed using the title and abstract after duplicates (n = 290) were eliminated. After 130 full-text papers failed to fulfill qualifying conditions, just 32 were left for further evaluation. Each of the three met the criteria for evidence synthesis analysis.

There were 22,789 (55.2%) male patients in the three trials we considered, totaling 41,274 individuals (the LS group had 5351 while the ES group had 35,923). All of the included studies were retrospective cohorts [15-17]. In the USA, two investigations were conducted [15,17], and the third was conducted in Korea [16]. In 2018, the first research was carried out [15,17], and the most recent was carried out in 2019 [16].

Patients who had surgery sooner had fewer overall problems, fewer hospital days, and lower medical expenses than those who had surgery later, according to the results after a year of follow-up. However, early surgery was associated with a higher prevalence of wound infections [15]. Despite incurring higher hospital stays and costs, the late surgery group benefited from a lower rate of ileocecectomy or right hemicolectomy. This group also tended to have fewer overall complications, a lower incidence of wound infections, and a shorter LOS compared to the early surgery group, indicating potential benefits in delaying the surgery under certain circumstances. Finally, one study [17] suggested that delayed surgery leads to higher charges, increased lengths of stay, and more complications. This reflects the complex decision-making involved in determining the timing of appendectomy, where delayed intervention might exacerbate the severity of appendicitis, leading to more intensive and costly care requirements (Tables 1, 2).

**Table 1:** Outcome measures of the included studies ES, early surgery; LS, late surgery; NM, not mentioned.

Study ID	Country	Study design	Sociodemographics	LOS (days)		Complications (%)		Main outcomes
				ES	LS	ES	LS	
Saluja et al., 2018 [15]	USA	Retrospective cohort	ES: 7708; LS: 1132; median age: 10.6; male patients: 5258 (59.5%)	5	8	4574 (59.3%)	745 (65.8%)	Patients who had early surgery experienced fewer overall problems, hospital days, and medical expenses after one year of follow-up, but they also had a greater prevalence of wound infections.
Kim et al., 2019 [16]	Korea	Retrospective cohort	ES: 200; LS: 1067; median age: 42.5; male patients: 676 (53.3%)	5.8	9.6	NM	NM	Despite a longer overall hospital stay and greater overall costs, the incidence of right hemicolectomy and ileocecectomy was decreased in the pEIS group, and there was a propensity for a shorter length of stay, a prevalence of wound infection, and a reduced incidence of overall problems.
Symer et al., 2018 [17]	USA	Retrospective cohort	ES: 28,015; LS: 3152; median age: 48.1; male patients: 16,855 (54.9%)	5	9	13,546 (48.4%)	2140 (67.9%)	Patients getting delayed surgery had higher charges, longer lengths of stay, and more problems, according to this population-level research of severe appendicitis.

**Table 2:** Risk-of-bias assessment using ROBINS-I ROBINS-I, Risk Of Bias In Non-randomized Studies - of Interventions.

Study ID	Bias induced by confusion	Bias in the participants' selection	Prejudice in how interventions are categorized	Prejudice in the classification of interventions	Bias brought on by incomplete data	Inaccuracy in result measurement	Selection bias in the reported results	All-around prejudice
Saluja et al., 2018 [15]	Moderate	Low	Low	Low	Low	Moderate	Low	Low
Kim et al., 2019 [16]	Moderate	Low	Moderate	Low	Low	Moderate	Low	Moderate
Symer et al., 2018 [17]	Moderate	Moderate	Low	Low	Low	Moderate	Low	Moderate

Discussion

This systematic review elucidates the complex landscape of surgical timing for complicated appendicitis, revealing a fundamental contradiction in the current evidence base. The central finding is the stark divergence in outcomes between the two large-scale US studies [15,17], which consistently demonstrated superior outcomes for early appendectomy (shorter length of stay, lower costs, fewer complications), and the single Korean study [16], which reported advantages for a delayed approach, including a reduced rate of major procedures like ileocecectomy.

The direct opposition between these findings necessitates an exploration of potential explanatory factors. The discrepancy may stem from critical differences in patient populations, such as variations in the severity of appendiceal inflammation (phlegmon vs. well-contained abscess) at presentation. Furthermore, differences in healthcare systems between the US and Korea, including protocols for initial non-operative management, criteria for hospital admission/discharge, and financial structures, could significantly influence length of stay and cost outcomes. The definition and reporting of complications may also not be consistent; for instance, one study might have emphasized major septic complications while another focused on surgical-site infections, leading to divergent overall complication rates. The inherent limitations of the included studies, primarily their retrospective design and the small final number of studies available for synthesis, prevent a definitive resolution of this conflict. This very limitation underscores that the current literature is inconclusive.

This analysis confirms the established definitions of the two intervention strategies. Early appendectomy, while lacking a single universal definition, is typically performed immediately or within the initial hospital admission [18,19]. In contrast, delayed (or interval) appendectomy involves a period of conservative management with antibiotics, often with percutaneous drainage, followed by scheduled surgery weeks later [18,20]. The clinical debate on the necessity of delayed appendectomy persists, with some experts advocating for its selective use only in cases of recurrent symptoms [18,20], while others maintain a different view [21].

Our findings align with the uncertainty noted in prior research. Cheng et al. [22] concluded that significant advantages or disadvantages of either approach could not be ruled out due to a lack of robust data. Conversely, other evidence, such as that from Calpin et al. [23], supports the superiority of intervention within 24 hours of admission.

Strengths and Limitations

A strength of this review lies in the large, real-world sample sizes of the included studies, which enhance the generalizability of the findings to clinical practice. However, these strengths are tempered by significant limitations. The inclusion of only three studies with conflicting results is the main limitation, along with the retrospective nature of the evidence introducing risks of selection bias and unmeasured confounding. Furthermore, the reliance on administrative data can lead to inconsistencies in outcome reporting. Most critically, the heterogeneity in defining "early" and "delayed" surgery complicates cross-study comparisons and limits the ability to draw unified conclusions.

## Conclusions

This systematic review highlights the complex and nuanced decision-making involved in the timing of appendectomy for complicated appendicitis. The synthesized evidence reveals a clear tradeoff: early surgery is consistently associated with a shorter overall hospital stay and lower costs but carries a higher risk of wound infections. In contrast, a delayed approach may reduce the need for major surgical procedures like hemicolectomy, as demonstrated in one study, but can lead to increased healthcare utilization in other settings.

Given the limited number of studies and their directly conflicting results, with two large US studies favoring early intervention and one Korean study favoring a delayed strategy, no definitive, universal recommendation can be made. The optimal timing is not a one-size-fits-all decision but should be individualized, weighing patient-specific factors, institutional resources, and surgical expertise.

To resolve this clinical equipoise, future research must prioritize large-scale, multicenter RCTs that adhere to standardized definitions for "early" (e.g., within 24 hours) and "delayed" (e.g., after 6-8 weeks of non-operative management) intervention. These studies should aim to identify specific patient subgroups (e.g., based on abscess size or comorbidities) that would benefit most from one strategy over the other, paving the way for personalized treatment protocols that optimize patient outcomes and resource allocation.
